# Sesquiterpene Lactones from *Vernonia cinerascens* Sch. Bip. and Their in Vitro Antitrypanosomal Activity

**DOI:** 10.3390/molecules23020248

**Published:** 2018-01-27

**Authors:** Njogu M. Kimani, Josphat C. Matasyoh, Marcel Kaiser, Reto Brun, Thomas J. Schmidt

**Affiliations:** 1 Institute of Pharmaceutical Biology and Phytochemistry (IPBP), University of Münster, PharmaCampus Corrensstraße 48, D-48149 Münster, Germany; m_kima01@uni-muenster.de; 2 Department of Chemistry, Egerton University, P.O. Box 536, Egerton 20115, Kenya; josphat2001@yahoo.com; 3 Swiss Tropical and Public Health Institute (Swiss TPH), Socinstr. 57, CH-4051 Basel, Switzerland; marcel.kaiser@unibas.ch (M.K.); reto.brun@unibas.ch (R.B.); 4 University of Basel, Petersplatz 1, CH-4003 Basel, Switzerland

**Keywords:** Asteraceae, sesquiterpene lactones, *Vernonia cinerascens*, *Trypanosoma brucei rhodesiense*, antitrypanosomal activity

## Abstract

In the endeavor to obtain new antitrypanosomal agents, particularly sesquiterpene lactones, from Kenyan plants of the family Asteraceae, *Vernonia cinerascens* Sch. Bip. was investigated. Bioactivity-guided fractionation and isolation in conjunction with LC/MS-based dereplication has led to the identification of vernodalol (**1**) and isolation of vernodalin (**2**), 11β,13-dihydrovernodalin (**3**), 11β,13-dihydrovernolide (**4**), vernolide (**5**), 11β,13-dihydrohydroxyvernolide (**6**), hydroxyvernolide (**7**), and a new germacrolide type sesquiterpene lactone vernocinerascolide (**8**) from the dichloromethane extract of *V. cinerascens* leaves. Compounds **3**–**8** were characterized by extensive analysis of their 1D and 2D NMR spectroscopic and HR/MS spectrometric data. All the compounds were evaluated for their in vitro biological activity against bloodstream forms of *Trypanosoma brucei rhodesiense* and for cytotoxicity against the mammalian cell line L6. Vernodalin (**2**) was the most active compound with an IC_50_ value of 0.16 µM and a selectivity index of 35. Its closely related congener 11β,13-dihydrovernodalin (**3**) registered an IC_50_ value of 1.1 µM and a selectivity index of 4.2.

## 1. Introduction

Human African trypanosomiasis (HAT or sleeping sickness), Chagas disease, and leishmaniases are some of the most neglected diseases among the eighteen neglected tropical diseases according to WHO [1,2]. The mentioned diseases are caused by protozoan parasites of the trypanosomatid family, order kinetoplastida, namely *Trypanosoma brucei* subsp., *Trypanosoma cruzi*, and *Leishamania* spp, respectively, which are transmitted by insect vectors [3,4,5,6]. Millions of people are affected so these diseases are a big public health concern, and more since the affected populations form a non-lucrative sector for drug development [5,6,7,8]. Moreover, the available drugs for the treatment of these infections, which were developed more than 50 years ago, are highly toxic, have a poor efficacy, and exhibit increasing drug resistance [4,5]. Therefore, new drugs are required; in particular, those that are appropriate for resource-limited rural health systems. Natural products have, in many instances, shown high activity against the mentioned parasites [9,10].

Plants of the family Asteraceae are a rich source of sesquiterpene lactones (STLs) [11,12,13,14,15,16,17], which have been reported to have a wide range of biological activity such as anti-inflammatory, cytotoxic, antiplasmodial, and antitrypanosomal, among others [18,19,20,21,22,23]. Previous work in our group has shown that STLs are good antiprotozoal agents, particularly against *T. brucei rhodesiense*, the causative agent of the East African form of HAT, and therefore, could be of interest for drug discovery [17,24,25,26]. Very recently, we reported on antitrypanosomal STLs from *Vernonia lasiopus* with vernolepin as the most active compound from this plant [15]. In continuation of the study on STLs from this interesting plant genus with potential antitrypanosomal activity, we have now investigated *Vernonia cinerascens* Sch. Bip. (Asteraceae, subfamily Cichorioideae, tribe Vernonieae), which is an erect, highly branched annual herb that is 0.3–2 m tall [27,28]. It is native to Africa (Sudan, Ethiopia, Uganda, Kenya, Tanzania, Angola, Zimbabwe, Botswana, Namibia, South Africa) and Tropical Asia (India) [27]. It is used ethnomedicinally to treat urinary tract infections, male sterility, internal ulcers, constipation, navel aches, and gastritis [29,30]. Hirsutinolide type STLs have previously been reported from this plant [30,31], in addition to vanillic acid, isoferulic acid, caffeic acid, methyl gallate, uridine, 3′-methylquercetin, and quercetin [32]. We report herein on the antitrypanosomal activity of the dichloromethane leaf extract of this plant and its isolated constituents.

## 2. Results and Discussion

The dichloromethane (CH_2_Cl_2_) extract obtained from leaves of *V. cinerascens* was tested for in vitro antiprotozoal activity against *Trypanosoma brucei rhodesiense* (*Tbr*) (STIB 900 strain) trypomastigotes, *T. cruzi* (*Tcr*) (Tulahuen C4 strain) amastigotes, *Leishmania donovani* (*Ldon*) (MHOM-ET-67/L82 strain) axenic amastigotes, and *Plasmodium falciparum* (*Pf*) (NF54 strain) intra-erythrocytic forms. It was also evaluated for in vitro cytotoxicity against mammalian cells, specifically the rat-skeletal myoblast cell line L6. The extract was highly potent against *Tbr* with an IC_50_ value of 0.24 µg/mL and moderately active against *Ldon* as well as *Pf* with IC_50_ values of 4.0 and 5.0 µg/mL, respectively. The extract exhibited low activity against *Tcr* with an IC_50_ value of 16.4 µg/mL, but was moderately cytotoxic with an IC_50_ value of 6.1 µg/mL against the L6 cell line. Therefore, the CH_2_Cl_2_ extract was fractionated by silica gel column chromatography and representative fractions were subjected to bioactivity testing against *Tbr* (STIB 900 strain) trypomastigotes. Fractions F5, F6, F8, and F9 showed IC_50_ values of 3.4, 1.4, 0.17, and 2.1 µg/mL, respectively.

The most active Fraction, F8, was analyzed by UHPLC/+ESIQTOFMS/MS and vernodalol (**1**), vernodalin (**2**), and 11β,13-dihydrovernodalin (**3**) were dereplicated (see Figure S1). These compounds had been isolated from *V. lasiopus* in our previous study and shown to have in vitro anti*Tbr* activity with IC_50_ values of 0.26 µM and 0.07 µg/mL for compound **1** and a mixture of compounds **2** and **3** (in the ratio of 2:1), respectively, which could not be further separated due to the low amount obtained [17]. In the present study, vernodalin (**2**) and 11β,13-dihydrovernodalin (**3**) could be isolated in an attempt to determine their individual bioactivity. Through preparative purification of F6 and F9, compounds **4**–**8** were obtained. The structures of all isolated compounds were unambiguously confirmed from their NMR spectroscopic data and HR/MS data (obtained by UHPLC/+ESIQTOFMS/MS analysis). Except for the new compound **8**, vernolide (**4**) [33], 11β,13-dihydrovernolide (**5**) [31,33], hydroxyvernolide (**6**) [31], and 11β,13-dihydrohydroxyvernolide (**7**) [31] were identified, and all analytical data obtained were in full agreement with those reported in literature.

**Figure 1:**
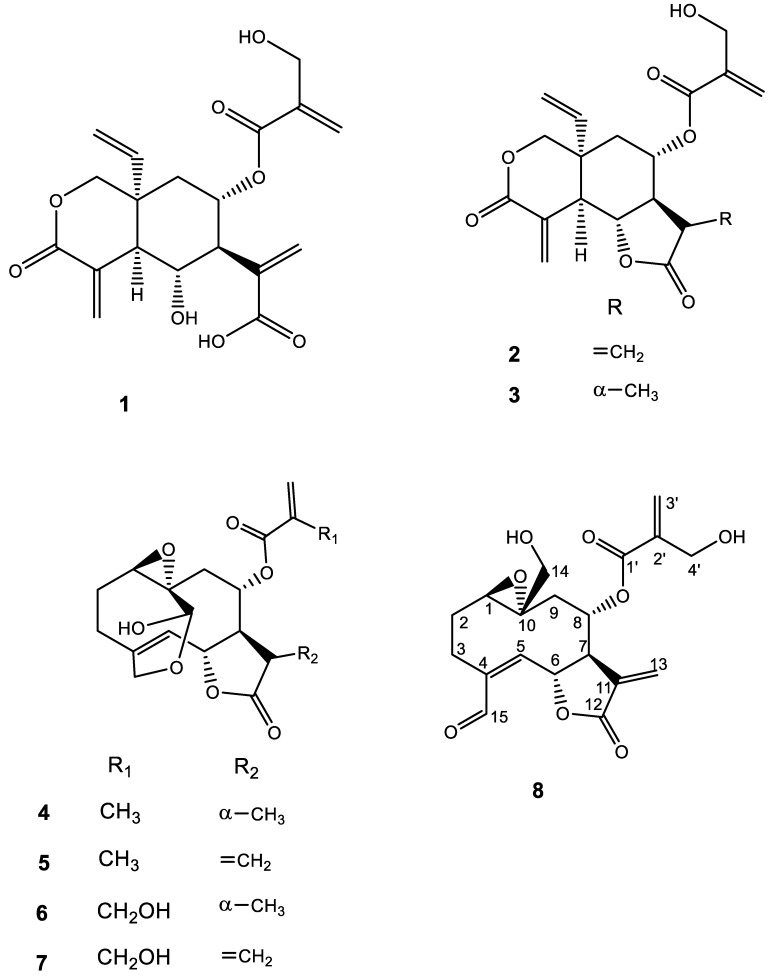
Sesquiterpene lactones isolated from *V. cinerascens*.

Vernocinerascolide (**8**) was obtained as a colorless amorphous solid and determined to have the molecular formula C_19_H_22_O_8_ by (+)-ESIQTOFMS/MS and was readily identified as a derivative of the germacrolide hydroxyvernolide **6** [28]. The ^1^H and ^13^C NMR spectra of **8** were very similar to those of **6**. However, in **8**, there was a carbonyl group (δ_C_ 195.9/δ_H_ 9.52) characteristic of a formyl functional group. The C-15 (δ_C_ 195.5) formyl group was assigned based on its HMBC correlations with H-3 and H-5 (Table 1). Moreover, HMBC correlations of H-15 (δ_H_ 9.52) with C-3 and C-4 were observed. Additionally, in **8**, there was an oxymethylene (δ_C_ 66.3/δ_H_ 4.07; 3.81) which replaced the doubly oxygenated C-14 methine in **6**. This indicated that the C-14-O bond on the C-14-O-C-15 bridge was cleaved in **8**. The β orientations of C-14, H-6, and H-8 were supported by NOESY correlations among H-14, H-6, and H-8. Similarly, the NOESY correlation between H-1 and H-7 affirmed the α orientation of H-1 and H-7. Thus, compound **8** was unambiguously assigned the depicted structure (Figure 1). We propose the generic name vernocinerascolide for this new germacrolide.

All isolated STLs were tested in vitro for activity against *Tbr*. Vernodalin (**2**) was the most active with an IC_50_ value of 0.16 µM and a selectivity index (SI) of 35. The 11β,13-dihydro derivative of vernodalin, **3**, had a reduced inhibition activity with an IC_50_ value of 1.1 µM. The reduction in activity can be attributed to the loss of one of the Michael acceptor systems upon hydrogenation of the C-11-C-13 exomethylene group. The Michael acceptors, α,β-unsaturated carbonyl groups, have been shown to alkylate biological macromolecules, thus inhibiting their normal functions [20,24,25]. These groups are generally held responsible for the high and wide range of biological activities of most STLs and it has been shown for other examples that the presence of more than one such system in the structure can dramatically enhance the activity [20,24,25]. The contribution of these groups to the biological activity of STLs is further corroborated by a comparison of the potency of vernolide (**5**) and 11β,13-dihydrovernolide (**4**), which had IC_50_ values of 0.50 and 17 µM, respectively. Similarly, hydroxyvernolide (**7**) and its congener 11β,13-dihydrohydroxyvernolide (**6**) registered IC_50_ values of 5.0 and 15 µM, respectively. The new germacrolide vernocinerascolide (**8**) had a moderate IC_50_ value of 4.8 µM and an SI of 27 (Table 2).

## 3. Materials and Methods

### 3.1. General Experimental Procedures

Optical rotation was measured on a JASCO P-2000 polarimeter (Groß-Umstadt, Germany). NMR spectra were recorded on a 600 MHz Agilent DD2 NMR spectrometer (Agilent Technologies, Santa Clara, CA, USA) at 298 K in CDCl_3_. The solvent signals (^1^H: 7.260 ppm and ^13^C: 77.000 ppm) were used to reference the spectra. MestReNOVA v. 11 (Mestrelab Research, Chemistry Software Solutions, Santiago de Compostela, Spain) software was used to process and evaluate the spectra. HRESIMS spectra were obtained on a UHPLC/+ESIQTOFMS/MS instrument with a Bruker Daltonics micrOTOF-QII quadrupole/time-of-flight mass spectrometer (Bruker Daltonics, Bremen, Germany) with an Apollo electrospray ion source operated in positive ionization mode. A comprehensive description of the UHPLC/+ESIQTOFMS/MS instrument settings and elution systems has been given elsewhere [17]. TLC was performed on precoated silica gel plates 60 F_254_ (Merck Chemicals GmbH, Darmstadt, Germany) with solvent systems consisting of hexane and ethyl acetate as the mobile phase. The plates were visualized under UV light at 254/360 nm and then sprayed with anisaldehyde/sulfuric acid reagent and heated on a hot plate. Column chromatography was performed on silica gel 60, 0.063–0.2 mm (Macherey-Nagel, Düren, Germany). Preparative HPLC isolations were carried out on a JASCO (Groß-Umstadt, Germany) preparative HPLC system (pump: PU-2087 plus; diode array detector MD 2018 plus; column thermostat CO 2060 plus; autosampler AS 2055 plus; LC Net II ADC Chromatography Data Solutions; sample injection loop: 2000 µL) on a preparative reversed-phase column Reprosil 100 C_18_ (5 µm, 250 mm × 20 mm, Macherey-Nagel, Düren, Germany) with binary gradients of the mobile phase consisting of water and MeOH.

### 3.2. Plant Material

The plant material of *V. cinerascens* was collected and identified in April 2016 in Narok (1.0768° S, 35.9533° E), Kenya, by S.T. Kariuki, a taxonomist at the Biological Sciences Department, Egerton University, Kenya. A voucher specimen has been deposited at the Institute of Pharmaceutical Biology and Phytochemistry, University of Muenster, Germany (voucher number Kimani, 04). The sample was dried in the shade at ambient temperature to constant weight and then ground.

### 3.3. Extraction and Isolation

The powdered plant material (600 g) was exhaustively extracted with CH_2_Cl_2_ (4.5 L) in a soxhlet apparatus and evaporated under vacuum, yielding 20.34 g of crude extract. The extract was tested for in vitro activity against *Tbr*, *Tcr*, *Ldon*, and *Pf*. The extract was chromatographed on a silica gel column (800 g) and eluted with hexane (2 L) to afford hexane fraction. The column was further eluted with hexane-EtOAc (3:2, 1:1, 3:7, 1:2, 0:10, *v*/*v*) to yield nine fractions (F1–F9). The fractions were analyzed by UHPLC/+ESIQTOFMS/MS and representative fractions subjected to bioactivity testing. Fraction F6 (86.3 mg) was separated by preparative HPLC with an optimized mobile phase composed of water (A) and methanol (B) using the following binary gradient conditions: 50% of B (0–5 min), 50–55% of B (5–10 min), 55–60% of B (10–15 min), 60–70% of B (15–20 min), 70–80% of B (20–25 min), 80–100% of B (25–30 min) which was held for 5 min, 100–50% of B (45–40 min) which was held for a further 4 min, and a flow rate of 9 mL/min was maintained. This yielded compounds **4** (3.7 mg; *t*_R_: 23.83–24.90 min) and **5** (6.4 mg; *t*_R_: 25.23–25.8 min). Upon analysis of fraction F8 by UHPLC/+ESIQTOFMS/MS, compounds **1**–**3** were dereplicated. In order to isolate compounds **2** and **3**, this fraction (99.2 mg) was separated using silica gel 60 column chromatography with hexane-EtOAc (3:7, *v*/*v*) as the mobile phase to yield two subfractions F8_1_–F8_2_. Subfraction F8_2_ (33.1 mg) was then separated by preparative HPLC using an optimized binary gradient mobile phase composed of water (A) and methanol (B) as follows: 40% of B (0–10 min), 40–45% of B (10–15 min), 45–50% of B (15–20 min) which was held for a further 5 min, 50–100% of B (25–30 min) which was held for 5 min, and 100–40% of B (45–40 min) which was held for 4 min. The flow rate was maintained at 10 mL/min. This yielded compounds **2** (4.2 mg; *t*_R_: 23.35–24.00 min) and **3** (3.7 mg; *t*_R_: 25.00–26.30 min). Similarly, the separation of fraction F9 (22 mg) by preparative HPLC, using an optimized binary gradient mobile phase composed of water (A) and methanol (B), occurred as follows: 40% of B (0–10 min), 40–45% of B (10–15 min), 45–50% of B (15–20 min) which was held for a further 5 min, 50–100% of B (25–30 min) which was held for 5 min, and 100–40% of B (45–40 min) which was held for 4 min at a flow rate of 10 mL/min, gave compounds **6** (3.7 mg; *t*_R_: 14.03–15.10 min), **7** (3.5 mg; *t*_R_: 11.57–12.93 min), and **8** (2.8 mg; *t*_R_: 9.13–9.84 min). 

### 3.4. Analytical Data

Vernocinerascolide **8**: Colorless armophous solid; ${\left[\alpha \right]}_{D}^{18}$ +28 (*c* 0.1, MeOH); ^1^H and ^13^C NMR (CDCl_3_), see Table 1; (+)HRESIMS *m*/*z* 379.3822 [M + H]^+^ (calcd for C_19_H_23_O_8_, 379.1393); 396.4218 [M + NH_4_]^+^ (calcd for C_19_H_26_NO_8_, 396.1658); 401.3785 [M + Na]^+^ (calcd for C_19_H_22_O_8_Na, 401.1212); UHPLC/+ESIQTOFMS *t*_R_: 5.33 min.

### 3.5. In Vitro Bioassays

In vitro bioactivity tests were performed as previously described [24] against *Tbr* (bloodstream trypomastigotes, STIB 900 strain), *Tcr* (amastigotes, Tulahuen C4 strain), *Ldon* (axenic amastigotes, MHOM-ET-67/L82 strain), and *Pf* (intraerythrocytic forms, NF54 strain), and the cytotoxicity test was conducted against mammalian cells (L6 cell line from rat-skeletal myoblasts) at the Swiss Tropical and Public Health Institute (Swiss TPH, Basel, Switzerland). 

## 4. Conclusions

In summary, the search for novel antiprotozoal agents from *V. cinerascens* is described in this work. The elemanolide type STLs vernodalol (**1**), vernodalin (**2**), and 11β,13-dihydrovernodalin (**3**) were dereplicated from the dichloromethane extract of *V. cinerascens* leaves. Moreover, isolation and the structure identification of a new germacrolide type STL vernocinerascolide (**8**), together with four known compounds (**4**–**7**) of a similar type, are described. These compounds were obtained from the leaves of *V. cinerascens* for the first time. In addition, compounds **2**–**8** displayed strong or moderate antitrypanosomal activity against *Tbr* blood stream forms and compound **2** was the most active with an IC_50_ value of 0.16 µM and an SI value of 35.2. Due to this considerably high activity, this compound warrants further investigations such as in vivo and mechanism of action studies.

## Figures and Tables

**Table 1:** NMR spectroscopic data of compound **8** (150 and 600 MHz, CDCl_3_).

Position	δ_C_	δ_H_ (multi., *J* in Hz)	HMBC
1	64.5, CH	2.75, dd (9.8, 4.9)	2, 3, 10
2	28.4, CH_2_	α 2.24, ddt [tt] (13.9, 4.8)	3, 10
		β 1.58, m	
3	23.37, CH_2_	2.51, td (13.5, 4.8)	1, 2, 4, 5, 15
4	144.9, C		
5	149.3, CH	6.35, dd (10.6, 1.0)	3, 4, 7, 15
6	76.8, CH	6.10, dd (10.6, 1.5)	4, 7, 8, 11, 12
7	52.7, CH	3.01, ddd [dq] (9.9, 1.7)	5, 8, 9, 11, 12, 13
8	72.3, CH	5.58, ddd (11.5, 9.9, 3.4)	1′
9	47.2, CH_2_	β 2.83, m	1, 7, 8, 10, 14
		α 1.43, ddd (13.2, 11.5, 1.4)	
10	60.0, C		
11	135.8, C		
12	171.2, C		
13	131.4, CH_2_	a 6.43, d (1.7)	7, 8, 11, 12
		b 5.78, d (1.6)	
14	66.3, CH_2_	4.07, dd (11.0, 0.8)	1, 9, 10
		3.81, dd (11.0, 1.4)	
15	195.9, CH	9.52, d (1.0)	3, 4
1′	167.4, C		
2′	141.8, C		
3′	128.9, CH_2_	a 6.21, dt [q] (0.9)	1′, 2′, 4′
		b 5.89, dt [q] (1.3)	
4′	64.9, CH_2_	4.29, dd [t] (1.1)	1′, 2′, 3′

**Table 2:** In vitro antitrypanosomal and cytotoxic activity IC_50_ values of the isolated compounds **2**–**8** *^a^*.

Compound	*Tbr* (µM)	Cytotoxicity (µM)	SI
**2**	0.16 ± 0.04	5.6 ± 0.0	35
**3**	1.1 ± 0.3	4.7 ± 0.4	4.2
**4**	17 ± 0	13 ± 2	0.8
**5**	0.50 ± 0.01	6.9 ± 0.0	13
**6**	15 ± 0	49 ± 1	3.2
**7**	5.0 ± 0.0	22 ± 1	4.3
**8**	4.8 ± 1.1	128 ± 1	27
PC *^b^*	0.003 ± 0.001	0.007 ± 0.001	

*^a^* Data are means of two independent determinations ± absolute deviation; for activity of compound **1**, see [15]. *^b^* PC—Positive control: melarsoprol (*Tbr*), and podophyllotoxin (cytotox. L6).

## Supplementary Materials

The UHPLC/+ESIQTOFMS/MS chromatograms and spectra of compounds **1**–**3** and **8**, and 1D and 2D NMR spectra of compound **8** as supplementary Figures S1–S8 available online.
