# Supplementary material for: Sesquiterpene Lactones from Vernonia cinerascens Sch. Bip. and Their in Vitro Antitrypanosomal Activity
Source: Molecules. 2018 Jan 27;23(2):248. doi: 10.3390/molecules23020248 (PMC6017816; doi:10.3390/molecules23020248)
Supplement: Supplementary file 1 [file molecules-23-00248-s001.pdf]

Supplementary material

Sesquiterpene lactones from *Vernonia cinerascens* Sch. Bip. and their *in vitro* antitrypanosomal activity

Njogu M. Kimani[1], Josphat C. Matasyoh[2], Marcel Kaiser[3,4] Reto Brun[3,4] and Thomas J. Schmidt[1,*]

[1] Institute of Pharmaceutical Biology and Phytochemistry (IPBP), University of Münster, PharmaCampus Corrensstraße 48, Münster D-48149, Germany; Email: m_kima01@uni-muenster.de

[2] Department of Chemistry, Egerton University, P.O. Box 536, Egerton 20115, Kenya; Email: josphat2001@yahoo.com

[3] Swiss Tropical and Public Health Institute (Swiss TPH), Socinstr. 57, Basel CH-4051, Switzerland; Email: marcel.kaiser@unibas.ch (M.K.); reto.brun@unibas.ch (R.B.)

[4] University of Basel, Petersplatz 1, Basel CH-4003, Switzerland

* Correspondence: thomschm@uni-muenster.de; Tel.: +49-251-83-33378

Compounds **1-3**

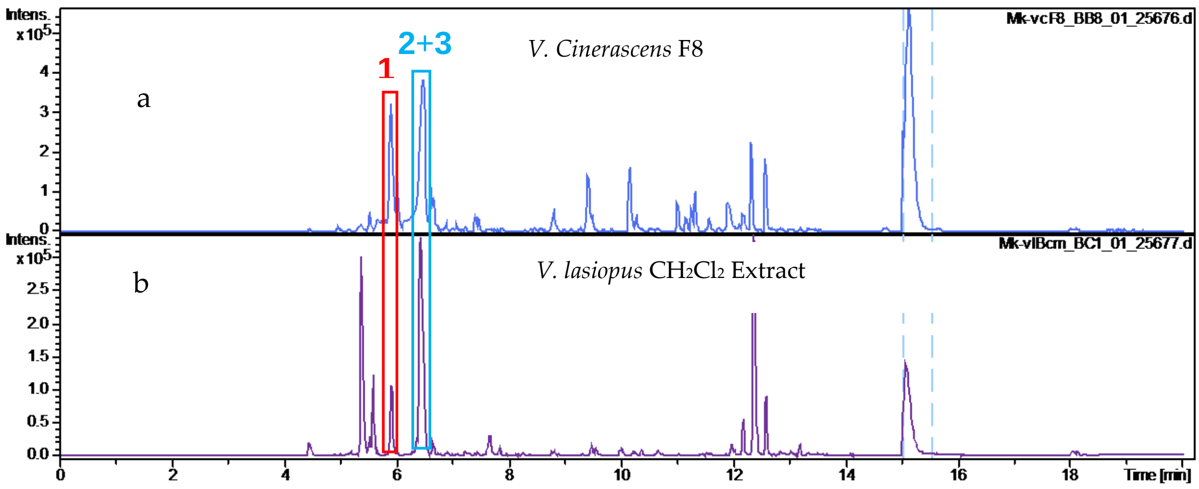

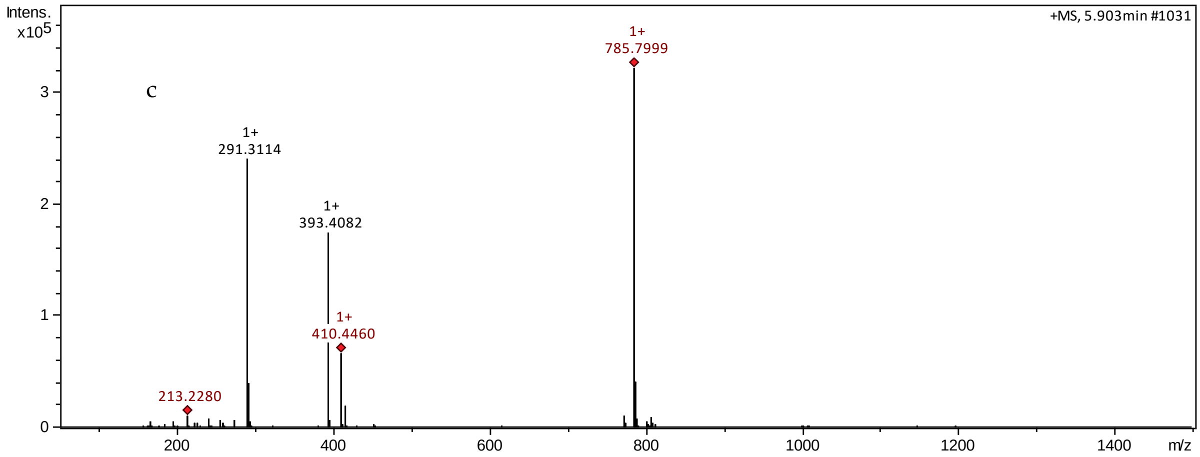

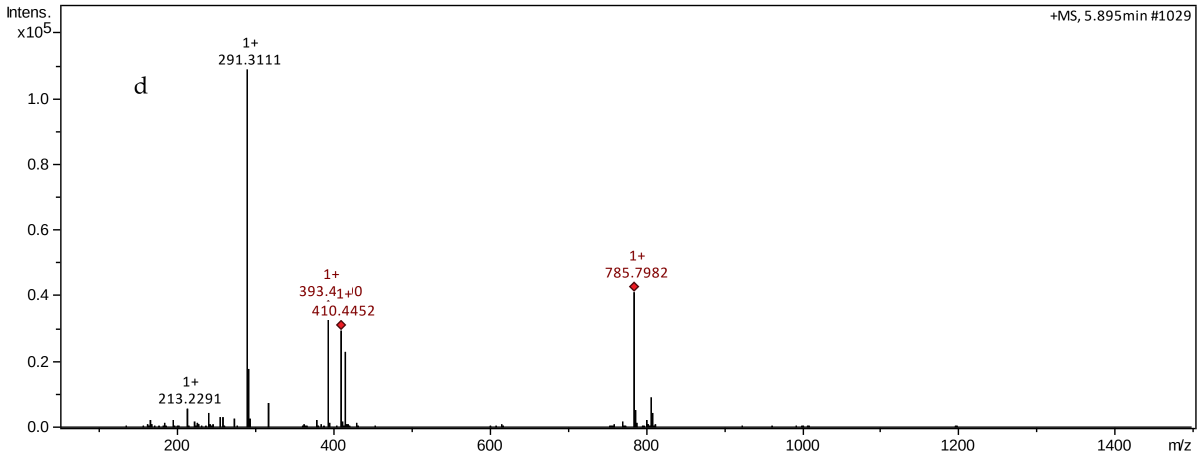

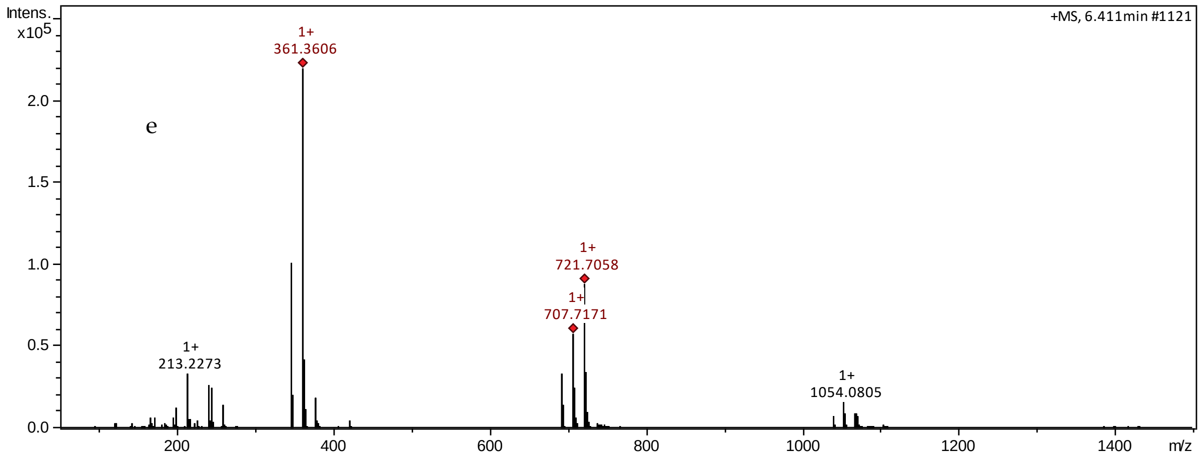

Intens.
x10^5
+MS, 6.411min #1121
e
1+
361.3606
1+
721.7058
1+
707.7171
1+
213.2273
1+
1054.0805
m/z

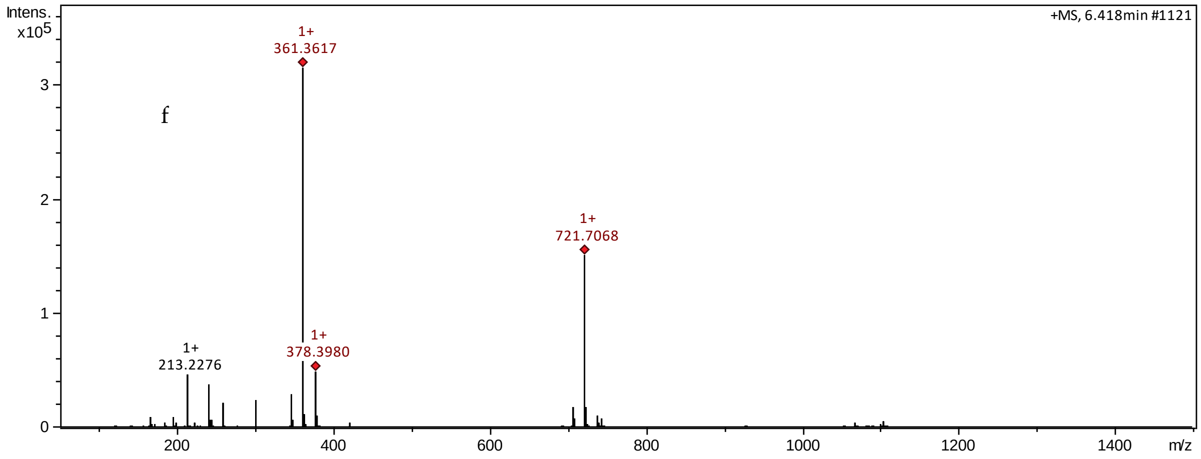

Intens.
x10^5
+MS, 6.418min #1121
f
1+
361.3617
1+
721.7068
1+
213.2276
1+
378.3980
m/z

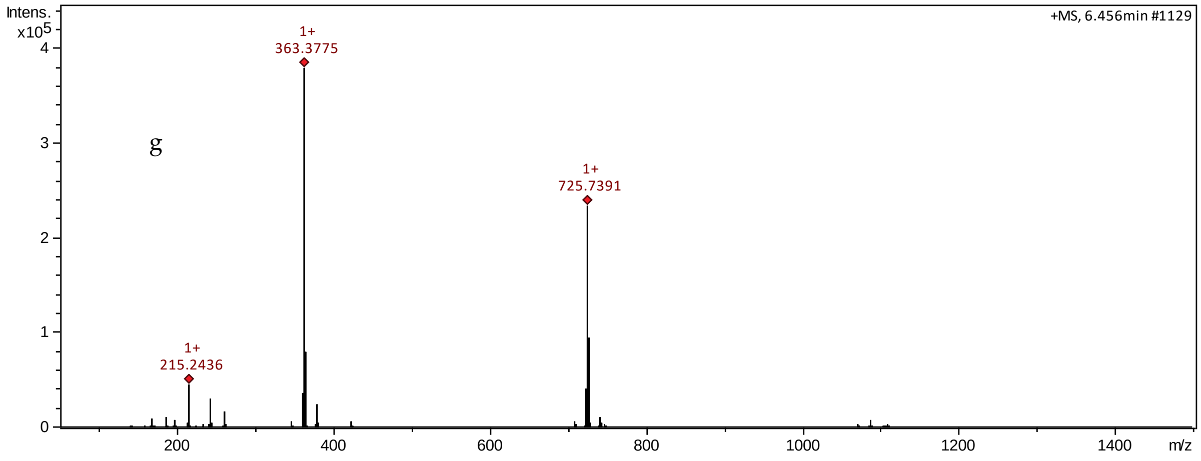

Intens.
x10^5
+MS, 6.456min #1129
g
1+
363.3775
1+
725.7391
1+
215.2436
m/z

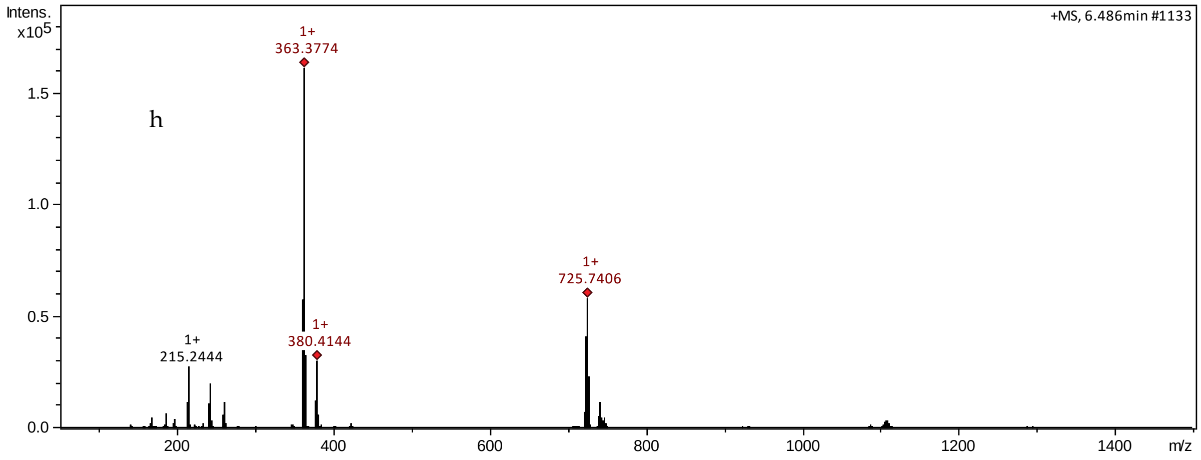

Figure S1: UHPLC/+ESIQTOFMS/MS Chromatograms and Spectra of Compounds **1-3**. [a]+ESIQqTOFMS chromatogram of *V. cinerascens* fraction F8; [b]+ESIQqTOFMS chromatogram of *V. lasiopus* CH_2Cl_2 extract; [c]+ESI/MS spectrum of Compound **1** from *V. cinerascens* fraction F8. [d]+ESI/MS spectrum of Compound **1** from *V. lasiopus*. [e]+ESI/MS spectrum of Compound **2** from *V. cinerascens* fraction F8. [f]+ESI/MS spectrum of Compound **2** from *V. lasiopus*. [g]+ESI/MS spectrum of Compound **3** from *V. cinerascens* fraction F8. [h]+ESI/MS spectrum of Compound **3** from *V. lasiopus*.

Compound **8**

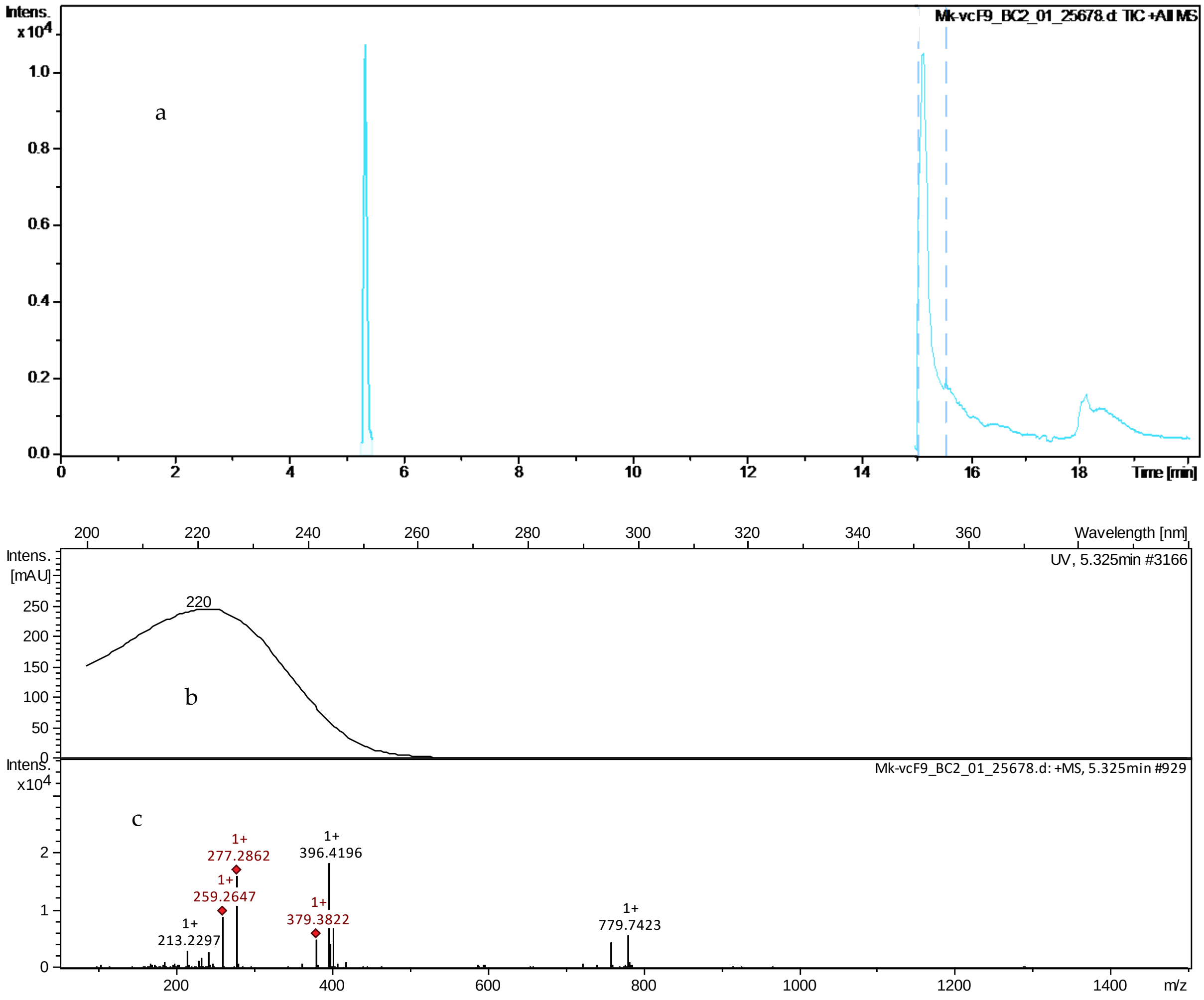

Figure S2: UHPLC/+ESIQTOFMS/MS Chromatogram and Spectra of Compound **8**. [a]+ESIQqTOFMS chromatogram; [b]UV spectrum; [c]+ESI/MS spectrum.

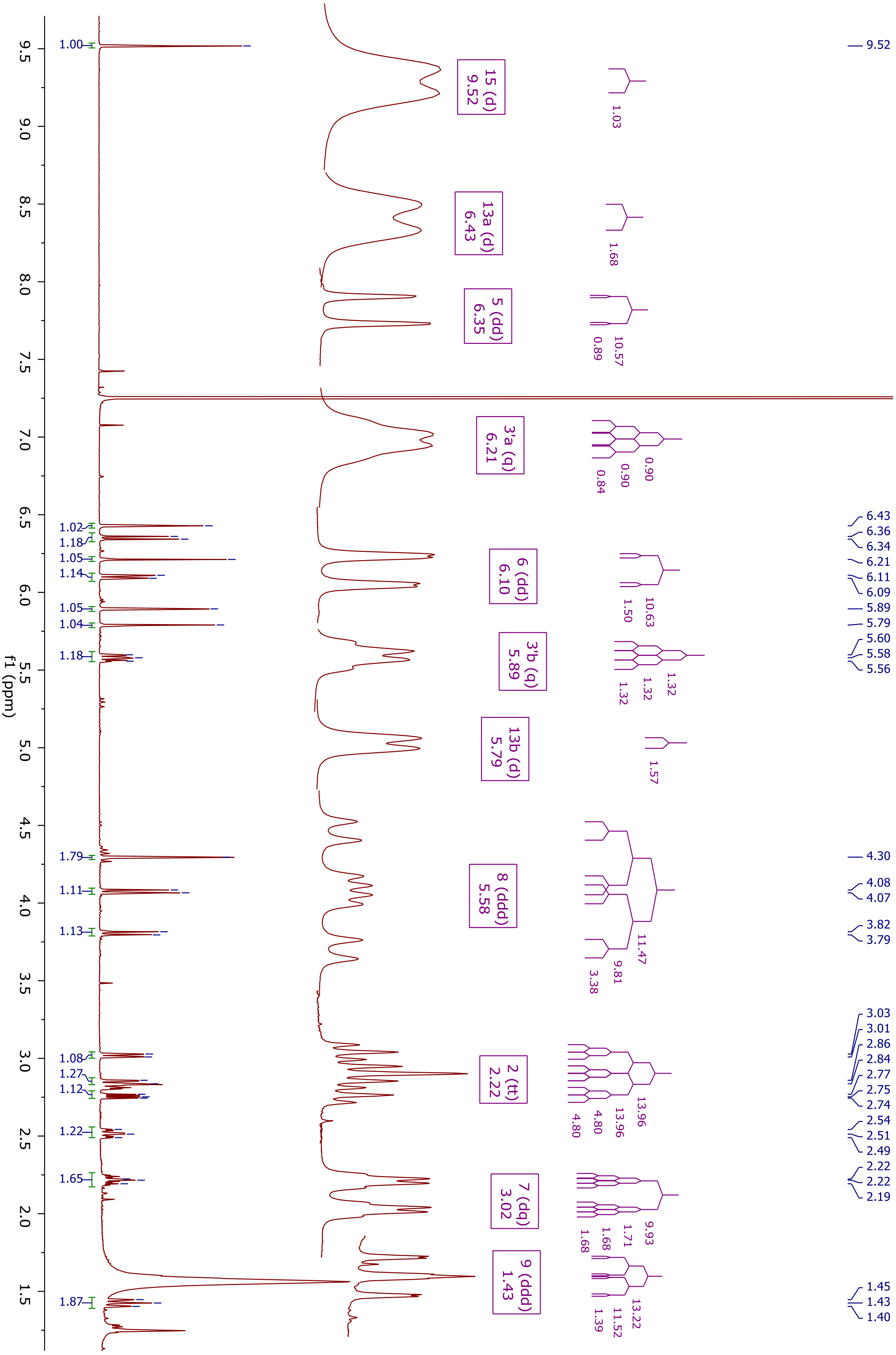

Figur S3: ^{1}H NMR ($CDCl_3$, 600 MHz) spectrum of compound **8.** Please note that the peak annotations at the top apply to the full spectrum shown at the bottom.

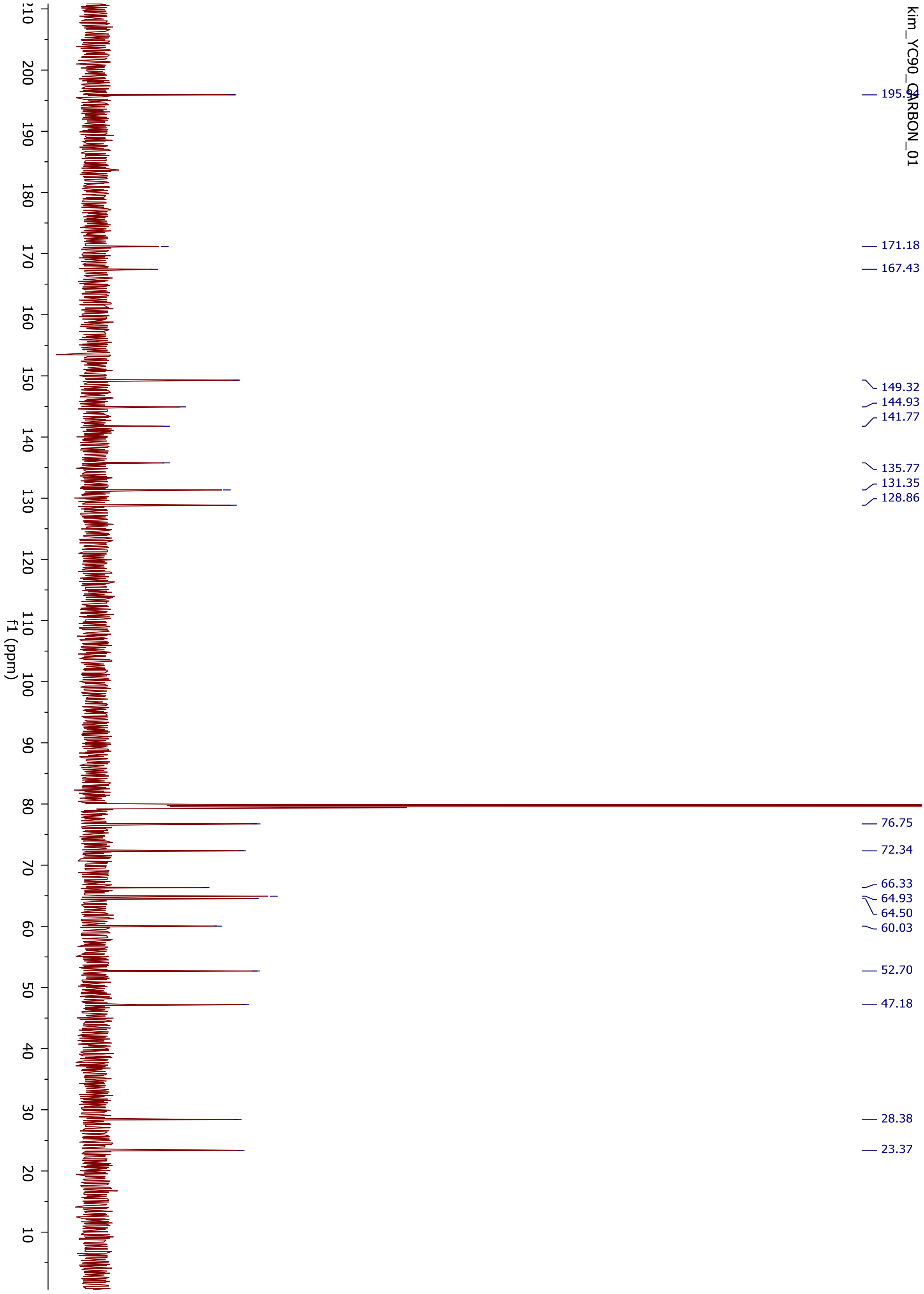

Figure S4: ^{13}C NMR ($CDCl_3$, 150 MHz) spectrum of compound **8**

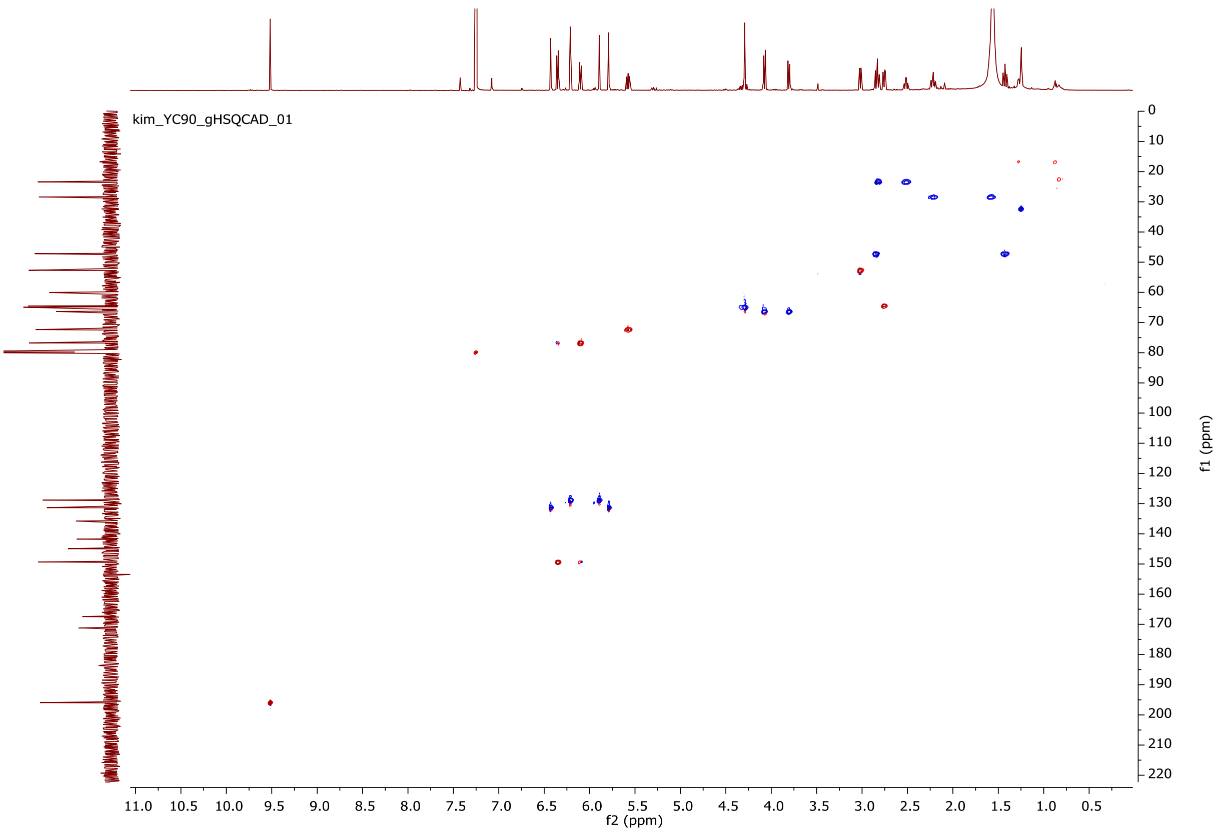

Figure S5: HSQC NMR ($CDCl_3$, 600 MHz) spectrum of compound **8**

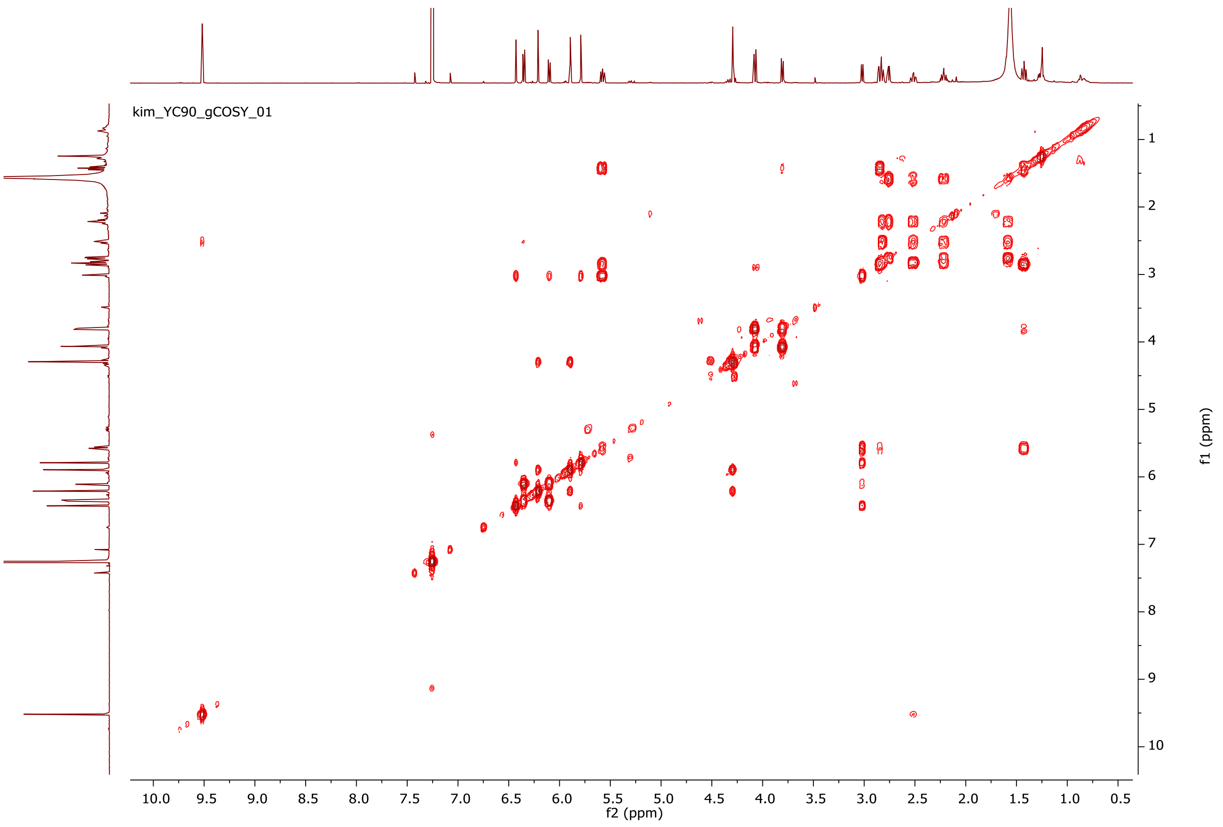

Figure S6: ^{1}H-^{1}H COSY NMR ($CDCl_3$, 600 MHz) spectrum of compound **8**

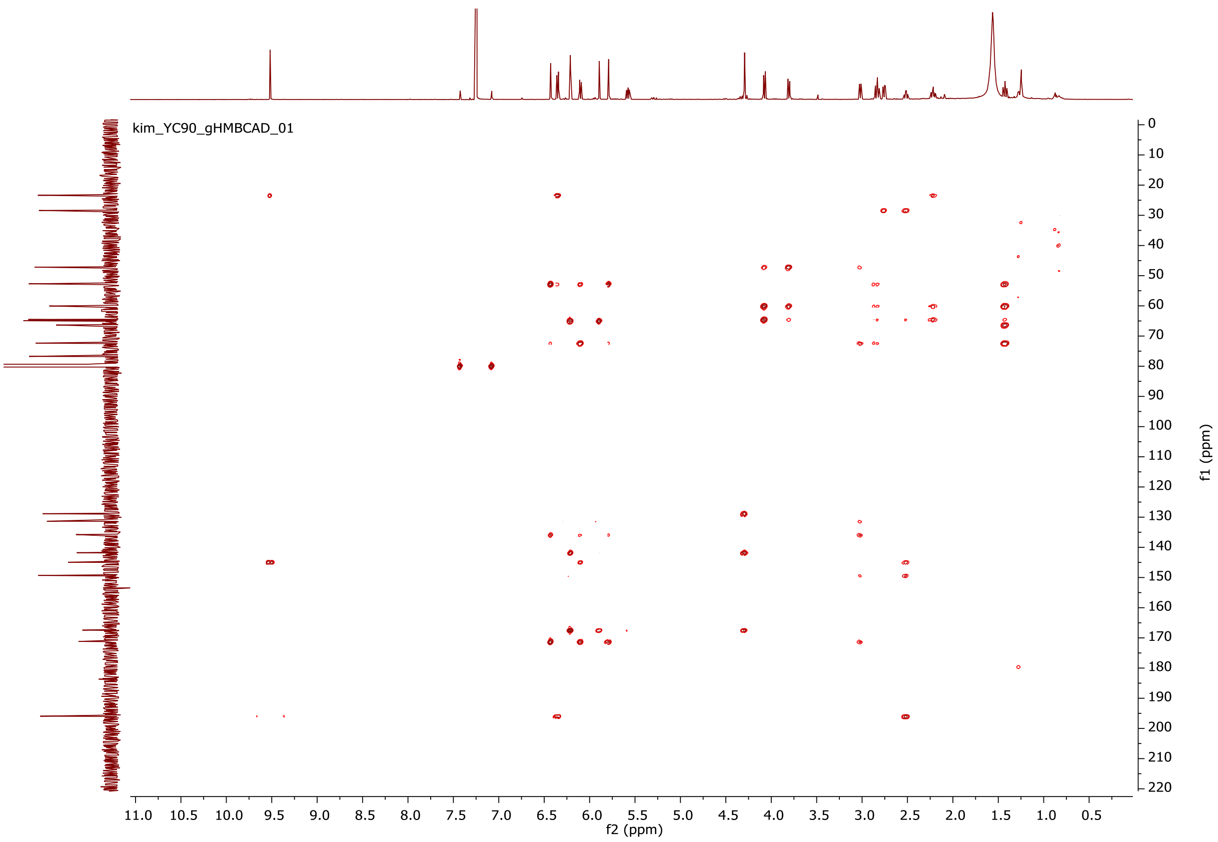

Figure S7: HMBC NMR ($CDCl_3$, 600 MHz) spectrum of compound **8**

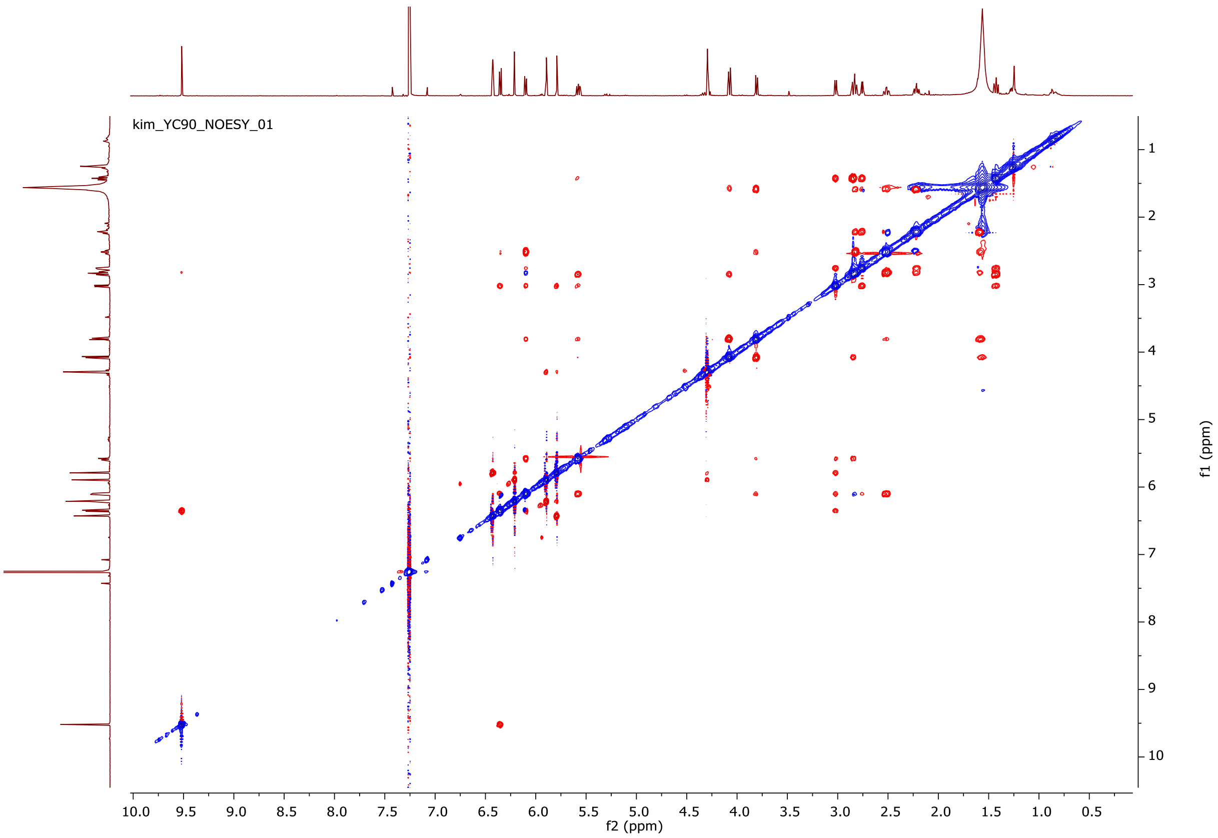

Figure S8: 2D NOESY NMR ($CDCl_3$, 600 MHz) spectrum of compound **8**
